# Localized pigmented villo-nodular synovitis of trochanteric bursa

**DOI:** 10.3205/iprs000178

**Published:** 2023-10-13

**Authors:** Juergen Bruns, Benedikt Rosenbaum, Christoph Thorns

**Affiliations:** 1 Dept. of Orthopedic Surgery, Wilhelmsburg Hospital Groß-Sand, Hamburg, Germany; 2 Dept. of Radiology, Wilhelmsburg Hospital Groß-Sand, Hamburg, Germany; 3 Dept. of Pathology, Marienkrankenhaus Hamburg, Germany

**Keywords:** pigmented villo-nodular synovitis, PVNS, tenosynovial giant cell tumors, TSGCT, circumscribed, diffuse, bursa, bursa trochanterica

## Abstract

This is the first report on a localized pigmented villo-nodular synovitis (PVNS or TSGCT) occurring in the trochanteric bursa. Bursal involvement in PVNS is extremely rare. Most often PVNS occurs either as a localized or diffuse lesion in a major synovial joint, such as the knee, ankle joint or hip joint. In principle, all synovial structures can be involved.

The case reported here is remarkable regarding the long period between the occurrence of the first symptoms and the final diagnosis as well as the age of the female patient (75 yrs).

Therapeutically a complete resection was performed in order to avoid recurrence. More then three years later the patient did well and there has been no evidence of recurrence yet.

## Abstract (de)

Dies ist der erste Bericht über eine pigmentierte villo-noduläre Synovitis (PVNS) vom circumscripten Typ in der Bursa trochanterica. Am häufigsten tritt die PVNS in Knie-, Sprung- und Hüftgelenken entweder als circumscripte oder diffuse Form auf. Im Prinzip kann die PVNS in allen synovialen Strukturen auftreten. In Bursen tritt die PVNS äußerst selten auf. Weniger als 25 Berichte gibt es bisher über das Auftreten einer PVNS in einer Bursa. Dies ist, soweit bekannt, der Erste über den Befall der Bursa trochanterica.

Auffällig bei diesem Fall ist, dass die Zeit zwischen dem Auftreten erster Symptome bis zur endgültigen Diagnosestellung mit 7 Jahren sehr lang und die Patientin mit 75 Jahren deutlich älter war als andere Patienten mit PVNS.

Therapeutisch erfolgte eine komplette operative Resektion der Bursa trochanterica. Nach dreieinhalb Jahren postoperativ ist die Patientin frei von Symptomen und Rezidiven.

## Case description

### Preoperative findings

A 75-year-old female had been suffering from a fluctuating soft tissue mass on her lateral proximal thigh for seven years. Prior to the symptoms she had fallen on her left trochanter. Primary treatment was done symptomatically. Due to the long-lasting symptoms a MRI was finally performed exhibiting the findings mentioned below. The patient was admitted without any clinical signs of infection, an almost normal gait-pattern and a free range of motion of the ipsilateral hip joint. White blood cells (WBC) and C-reactive protein (CRP) were within normal range. 

### Imaging

**Figure 1:**
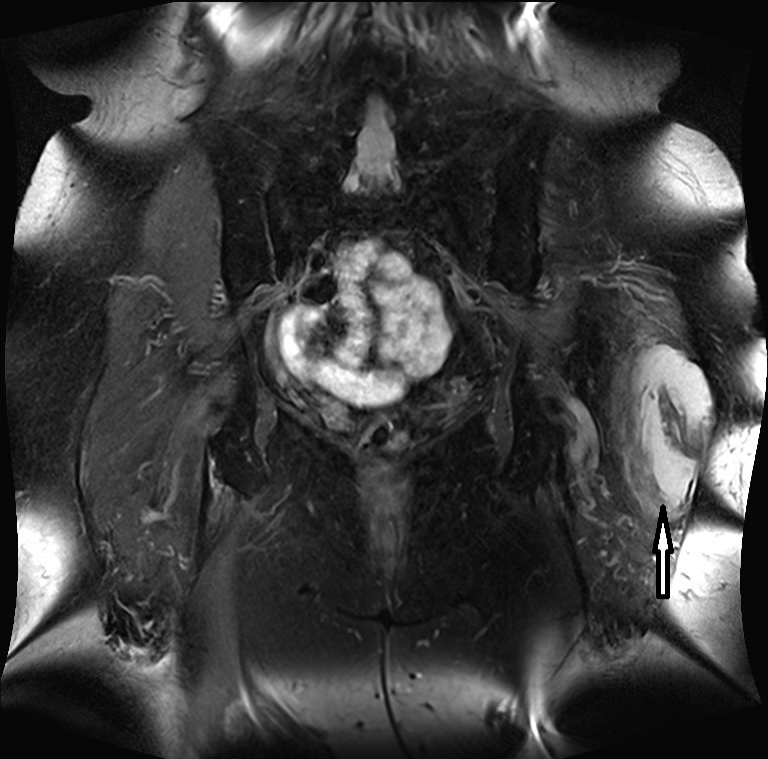
Proton-weighted coronal plane showing an enlarged bursa trochanterica on the left side

**Figure 2:**
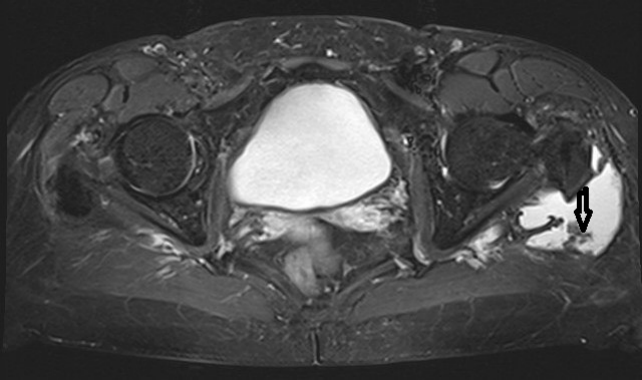
Proton-weighted transversal plane exhibiting the enlargd bursa filled with fluid and soft-tissue protruded into the bursa trochanterica (marked by an arrow)

**Figure 3:**
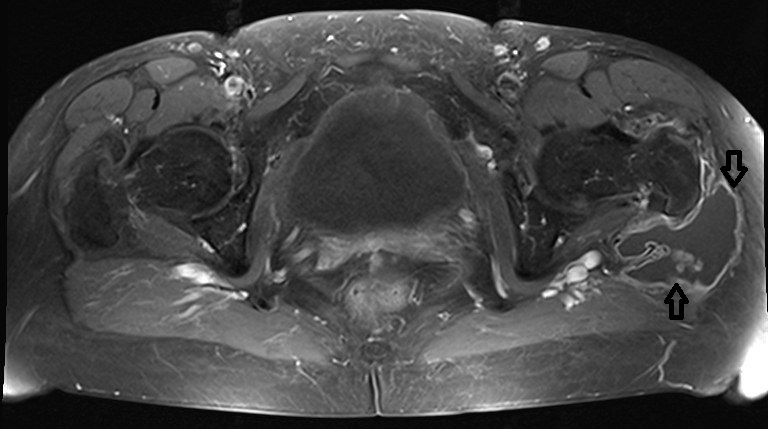
T-1 weighted fat-suppressed transversal plane with contrast medium exhibiting the enlargd bursa filled with fluid and soft-tissue protruded into the bursa trochanterica and surrounded by contrast medium (marked by the arrows)

**Figure 4:**
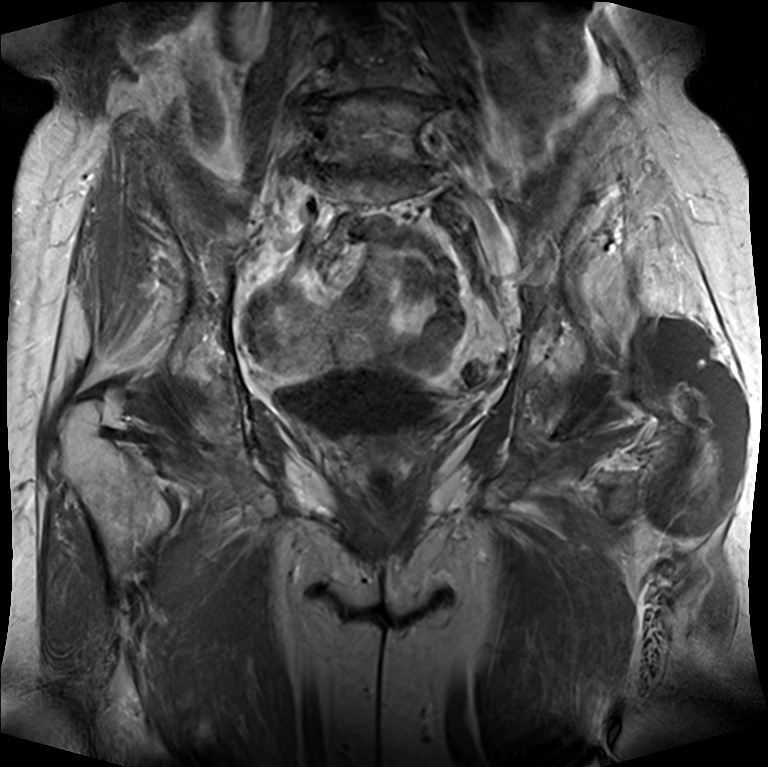
T-1 native coronal plane exhibiting the enlarged bursa trochanteria containing PVNS-soft-tissue

Lateral to the greater trochanter in the bursa trochanterica there was evidence of fluid showing homogeneous hyperintensity without sedimentation in fluid-sensitive sequences. Proton-weighted imaging revealed two nodular wall extensions in teardrop shape extending craniolaterally. These extensions measured a maximum of 1 cm at the base. In addition, at the roof of the fluid-filled cavity there was a space measuring approximately 1.5 x 0.5 cm. In the native fat-suppressed T1 turbospinecho sequence, this space-occupying lesion was partially hyperintense. This was considered to be hemosiderin. After application of gadolinium, there was a vigorous enhancement of the lining of the height. In addition, there was diffuse enhancement of the muscles in the immediate vicinity of the bursa. The hemorrhaged mass showed no enhancement of the contrast medium. In summary, the recent MRI exhibited an enlarged trochanteric bursitis with some intrabursal soft tissue formation suspicious for pigmented villo-nodular synovitis (PVNS) of the bursa trochanteric (PVNSBT) (Figure 1 (Fig. 1), Figure 2 (Fig. 2), Figure 3 (Fig. 3), Figure 4 (Fig. 4)). 

### Intraoperative findings

The enlarged trochanteric bursa exhibited several lacunae with a thickened synovial layer in terms of an unspecific synovitis and parts showing villo-nodular yellowish to brownish synovitic changes to the extensions already visable in the MRI. Furthermore, the medial glutaeus muscle exhibited degenerative lipomatous changes, probably as a sequela of her previous trauma. Therapeutically a complete resection of the trochantic bursa was performed to ensure total resection of PVNSBT tissue. Histologically PVNS of the bursa was confirmed without any signs of malignancy. Postoperatively the patient recovered well. 

### Follow-up

At final follow-up three and a half years postoperatively, no signs of recurrence could be detected with a follow-up MRI. Clinically the patient exhibited a slight limping owing to the weakness of the medial glutaeus muscle. 

## Discussion

Pigmented villo-nodular synovitis (PVNS) and/or giant-cell tumors of tendon sheaths / tenosynovial giant cell tumors (TSGCT) are rare diseases of synovial tissue occurring most often in synovial joints such as knee and ankle joints [1], [2], [3], [4], [5]. 

Apart from synovial joints in principle all synovial structures [6] such as teno-synovial tissue and bursa can be involved. Sometimes the synovial origin of the tumor is unclear: for example in the supraclavicular fossa [7], infratemporal fossa [8], quadriceps muscle [9], subcutaneous thigh [10], or unknown [6].

The incidence is reported to be about 1–2/million [11], [12], [13]. A more recent analysis exhibited a distinctly higher incidence: based on patients from the Netherlands Maastboom et al. [14] estimated the worldwide incidence: in their analysis digits were involved most often [29/million], followed by localized TSGCT in the extremities [10/ million] and 4/million for the diffuse type [14]. Furthermore, they found that the recurrence rate in diffuse types was 2.6 times higher compared to localized PVNS in the extremities.

Jaffe et al. [15] were, to our knowledge, the first to report on this particular disease. There were several synonyma, but meanwhile the terms teno-synovial giant-cell tumor (TSGCT) and/or villo-nodular synovitis (PVNS) are widely used [16]. Two different types of occurrence can be differentiated: the circumscribed type and the diffuse one [17]. 

PVNS is mostly an intraarticular process but may also expand towards the extraarticular vicinity of the involved joint. Other joints such as shoulder and elbow joint, the wrist, fingers, subtarsal joint, other joints of the foot, the temporo-mandibular and the infratemporal fossa [8], [16], [17], [18], [19] are rarely involved. An extremely rare occurrence was seen in joints of the vertebral column, mostly the facet joints but also even the iliosacral area and the sacrum [20], [21], [22], [23], [24].

Due to the osteolytic capability of giant-cells in PVNS the tumor can invade bone and produce osteolytic lacunae in these bones [25], [26], [27], [28] or even invade other anatomical bony structures as reported by Son et al. [29], who treated a diffuse PVNS located in the temporal area invading the skull followed by destruction of the brain parenchyma. In single case reports rare locations such as an intramuscular lesion of the quadriceps muscle or even a subcutaneous location far away from any synovial structure have been reported as well as PVNS tissue occurring in the external auditory canal [9], [10], [30]. 

Rarely, a multilocular articular occurrence is reported [31], [32]. In addition, similar to giant cell tumors of bone [33] even benign PVNS lesions can metastasize to regional lymph-nodes or to the lung [34], [35], [36]. A simultaneous appearance of PVNS with synovial chondromatosis of the hip has been reported by Efrima et al. [37]. 

Even in metastasing cases the primary lesions may normally exhibit no signs of malignancy. Benign but aggressive PVNS is mostly seen in adults aged between 20 and 50 years, but even in children this lesion has been found [13], [38], [39], [40]. In contrast, primary malignant PVNS is extremely rare. Fewer than 20 cases have been reported as yet [24], [41], [42].

The occurence in bursa has been described only once so far [43]. Only case reports on involved bursa have been reported (see below). In a binational analysis on 173 patients suffering from PVNS or TSGCT a bursal involvement was seen in only 4% of either the diffuse or localized type [43]. In principle, all bursa around the acromium, olecranon, iliopsoas muscle, fingers, toes, temporomandibular region and elsewhere can be affected [15], [44], [45], [46], [47]. The one involved most often was the anserine bursa [45], [46], [47]. Other bursae such as the suprapatellar, subacromial and ileopectinal bursa are rarely involved [48], [49], [50]. Others reported a case where PVS was located in a popliteal cyst [51], but anatomically this is not a true bursa. 

Apart from the hip joint there is only one report of PVNSB involving the iliopectineal bursa in the region of the hip [52]. 

Regarding the diagnostic procedure, the most important issue seems to be to remember such a rare disease. In addition, this case showed two specialities: the age of the patient was very high and the bursa trochanteric, normally a very small bursa, was extremely enlarged and contained “normal” infected synovial tissue parallel to PVNS synovitis with the typical macroscopic changes such as pigmentation, villi and noduli. The rarity of this location might have caused the long period between onset of symptoms and final diagnosis and immediate treatment. 

As for many other cases it is well-known that patients suffer for a long time before the final diagnosis is made as they experience unspecific symptoms such as tenderness, joint effusion, swelling and a limited range of motion. 

Regarding imaging procedures MRI has a central role in the identification of both the extra- and intraarticular tumors [53], [54]. PVNS mostly exhibits typical characteristics described above and in the literature [53], [54]. CT-scans might be helpful for the detection of a bony involvement in intra- and extraarticular diseases [55]. 

The current treatment of choice for both the extraarticular diffuse and localized type of PVNS as well as for TSGCT is the complete surgical removal of the tumor masses if possible [56], [57], [58]. 

In primary circumscribed tumors of either location (intra- or extrarticular) additional adjuvant therapeutical procedures such as external radiation or radiosynovectomy with isotopes do not seem to be indicated, and neither are new medical targeted therapies such as imitanib or similar medications.

In contrast, for the diffuse type an additional adjuvant therapy such as postoperative radiotherapy [1], [2], [59] or radiosynoviorthesis has been used at times in order to reduce the recurrence rate [60].

## Conclusion

PVNS is a very rare disease of synovial joints or other synovial structures containing synivial tissue such as tendon sheaths, and involvement of bursa is extremely rare. We report the first case involving the trochanteric bursa in a 75-year-old woman, who had suffered for seven years before being diagnosed. This indicates that the awareness of such a bursal lesion is most important to shorten the time until the final diagnosis is made. 

## Notes

### Competing interests

The authors declare that they have no competing interests.
